# Puerperal Insanity

**Published:** 1899-12-16

**Authors:** F. St. John Bullen


### Hospital Clinics and Medical Progress.

### PUERPERAL INSANITY.

By F. St. John Bullen, M.R.C.S.

The term puerperal insanity is often made to comprise the psychoses connected with the entire period of child-bearing, childbirth, and lactation, but should properly be confined to the mental disturbances occurring during labour and a certain time subsequently, which time may be more or less arbitrarily fixed. It is, admittedly, impossible to draw any strict line of demarcation between the insanity of this period and that preceding or following it; the slowly-developing mental disorder of later pregnancy may be precipitated into acute insanity by the added strain of labour, or the latter and unfortunate sequelæ may gradually trend towards the period of lactation, to be finally determined under that group. But, whilst recognising all this, there are many cases which justify the appellation of puerperal insanity from their independence of previous mental trouble and direct relation to the stress connected with the parturient and succeeding period. Such cases are considerably more frequent than those of the psychoses of pregnancy and lactation; they constitute very nearly half of the total. As regards the relative occurrence of puerperal insanity amongst all forms of insanity in women, the mean of numerous statistics gives this at about 8 per cent., and as to the proportion of it to deliveries in sane females, 1 in 400 would probably be a low estimate.

There are very many cases of puerperal mental disorders which for various reasons, such as the mild and transient nature, fear of social stigma, &c., never find their way into institutions for the insane, hence the asylum physician sees for the most part the more severe types of the malady. A definition of puerperal insanity must first be given. It is, briefly, a mental derangement which develops, or culminates, at the completion of labour, or which shows itself within a certain period after this. The time limit varies, and must be considered variable until a better classification of the different clinical forms grouped under the title of puerperal insanity can be made. It cannot be extended reasonably beyond six weeks from delivery, and in the majority of cases can be narrowed to about half this time. The percentage of cases, then, in which the onset of mental symptoms occurs within the first week of childbirth is stated at from 36 to 50 per cent.; within the fortnight, at 40 to 80 per cent.; and within the month, at 66 to 98 per cent. The higher percentages are in each instance those compiled from the most modern authorities. Thus it will appear that an estimate of four-fifths of the total cases as occurring within the first 14 days is assumable, and some authors have adopted this period as almost a definite limit to invasion by puerperal insanity proper. To be yet more exact, in the larger proportion of patients treated in asylums, the attack commences at from the fifth to the tenth day. The form of transitory frenzy which may accompany birth, or the transient delirious state following it, are not here included.

**The Etiology of Puerperal Insanity.**—There is here a complexity of more or less influential factors; some of these are of mild importance, but all need mention. Amongst the predisposing is age. Over half the total cases occur between the ages of 20 and 30. It may be assumed that, in proportion to the number of births occurring in women over 30 years of age, there is a greater relative risk of insanity with increasing age at the first pregnancy.

**Influence of First Childbirth.**—The danger of insanity is greater upon the first than subsequent pregnancies 38 per cent. of the total causes were primiparæ; the risk is most emphasised when the influence of later age (above 30 years) is added. The stress of the first pregnancy and birth, taken together with the often strong hereditary taint in puerperal insanity, is obviously sufficient to explain why the primipara readily succumbs. There are, however, especial dangers for the multiparæ; the influence of previous attacks, of perhaps rapid child-bearing, of increased domestic worries and privations, &c. Nearly 20 per cent. of the cases were recurrent. Some women, however, escape the troubl for the earlier pregnancies only to fall victims on a later one; the circumstances determining either the immunity in the earlier births or the breakdown in the later cannot be stated. The influence of hysteria is undecided; that it can only be for ill is certain, but it by no means follows that in a highly neurotic or hysterical subject puerperal insanity will occur. Excessive indulgence in alcohol has a greater effect when yielded to after labour than during pregnancy, but it does not form so important a factor in causation as might be supposed.

**Anæmia and Malnutrition.**—The rather sparse observations upon the blood in pregnancy and the puerperal state serve to show that anæmia is a common condition, and that there may be a very marked lessening of hæmoglobin and corpuscles; certain authors have found the number of red cells reduced to one-fifth or even less. Phthisis and exhausting diseases generally have more influence as the drain of lactation commences, but many cases of the melancholic form of puerperal insanity, which is later in developing than the maniacal, are phthisical, anæmic, or otherwise debilitated.

**Albuminuria** associated with eclampsia has, no doubt, a considerable tendency to predispose to mental disorder at or after parturition, but this conjunction only occurs in a small proportion of cases of puerperal insanity.

**Bad Heredity** exercises, as always, the paramount influence. In over 2,000 cases compiled by the older observers it is stated to be present in 37 per cent., whilst the average of several more modern returns, in which a larger recognition of hereditary influence is shown, is 49 per cent. And this may quite justifiably be regarded as an under-estimate for the same reasons that apply to the proportion of transmitted taint in insanity in general. It contrasts, nevertheless, in a marked fashion with the percentage of heredity for all forms of insanity. It seems probable that the predisposition is more strongly exercised through the female side of the family. That it is a weighty influence is evidenced by the frequent disproportion of the insanity as regards acuteness to the other exciting or predisposing causes, and by the breakdown after an often easy and natural labour.

**Exciting Causes.**—Under exciting causes are to be counted the moral and emotional. These include such circumstances as the shame of illegitimate birth, desertion of husband, especially shock from the death of the child, and various other causes. The first mentioned appears to have decided influence; according to the combined statistics of Batty Tuke and Clouston 21 per cent. of the cases of puerperal insanity occurred in unmarried women, this being thrice the proportion of illegitimacy in the general population of Edinburgh. As a whole, emotional and moral causes are reckoned as excitants in from 25 per cent. to 56 per cent. by various observers; if potent they will perhaps account for insanity without any pronounced heredity, it being remembered that during pregnancy the mental state of very many women is not quite normal. Where, however, the percentages vary so much as above, there must have been an imperfect recognition of the value of different causal conditions.

